# Response: Commentary: Consumer Reports of “Keto Flu” Associated With the Ketogenic Diet

**DOI:** 10.3389/fnut.2020.575713

**Published:** 2020-12-17

**Authors:** Emmanuelle C. S. Bostock, Kenneth C. Kirkby, Bruce V. Taylor, Jason A. Hawrelak

**Affiliations:** ^1^Menzies Institute for Medical Research, Hobart, TAS, Australia; ^2^Psychiatry, University of Tasmania, Hobart, TAS, Australia; ^3^Neurology, Menzies Institute for Medical Research, Hobart, TAS, Australia; ^4^College of Health and Medicine, University of Tasmania, Hobart, TAS, Australia; ^5^Australian Research Centre for Complementary and Integrative Medicine, University of Technology Sydney, Ultimo, NSW, Australia

**Keywords:** ketogenic diet, ‘keto flu', symptoms, online forums, scientific method

We thank the authors of the commentary on our paper *Consumer reports of “Keto Flu” associated with the Ketogenic Diet* (1) for sharing their concerns regarding the use of gray literature, in this case posts on online forums. As we read it, the central thesis of the commentary is that gray literature is inherently unreliable and should not be investigated unless there is a firmly grounded scientific understanding of the phenomenon. We do not fundamentally disagree with this proposition but regard the matter as undecided and wish to place discussion of the method in a broader context, as follows.

In our view the commentary does not address the main point, namely that for many health conditions and many putative treatments, there are a variety of narratives circulating in society. These typically include a scientific narrative and a consumer narrative, which may be broadly characterized as having a methodological and an experiential focus, respectively. We regard both as potentially “legitimate sources of information,” though either may not necessarily be a “source of legitimate information” on any given aspect. In other words, it is possible to accurately summarize both narratives, or aspects thereof, but whether they are true (Oxford English Dictionary: in accordance with fact or reality) or not is a different matter.

We contend that the scientific and consumer narratives are not mutually exclusive, and that both are subject to potential bias and may contain fraudulent or spurious material. Accordingly, we would rather see the study of consumer narratives incorporated as a standard component of the scientific agenda, the utility of this approach being determined on its merits. This would include the proviso that ethical considerations are met. This direction would complement moves to make scientific narratives available to consumers, for example through open access publishing, by rendering consumer narratives accessible to scientists.

Sharing, contesting, and informing the ideas in both the scientific and consumer narratives is the desired outcome.

With respect to specific issues raised in the commentary. The media report widely and with varying degrees of accuracy on many published journal articles and this possibility cannot govern the publication of results. There is a vast amount of consumer comment available through the internet and it would be impractical for interested parties to keep up with the full content. We believe that methods are required which accurately summarize this material with respect to the particular topic of enquiry. In our paper the topic was side effects of ketogenic diets (KD) and the focus within this category was in what is popularly termed “keto flu,” as discussed in online forums on this subject. Whether the aggregate of consumer comments in online forums is less reliable than conventional questionnaire-based methods is an empirical question. We have an open mind as to whether joining an online conversation, contemplating one's own experience and formulating this in writing in a form of public space is more or less reliable than scoring a questionnaire item or responding *ad-hoc* to a semi-structured interview question on a given side effect. It will be interesting to see how this field evolves.

The commentators have noted that ancillary information such as level of education and type of KD would assist in interpreting the results. We agree but have noted that for ethical reasons we did not analyse or report on personal information but rather confined our attention to the principal aim of discerning the pattern of side effects.

The analysis of gray literature is a growing area of research and the results of further studies will inform discussion of the merit and limitations of this approach. Between January and September 2020 twelve publications listed on PubMed reported analyses of online forum/ discussion board content. These covered a variety of topics, including irritable bowel syndrome (2), ketamine use in depression and post-traumatic stress disorder (3), and information seeking and sharing practices in breastfeeding mothers (4).

## Author Contributions

EB, KK, BT, and JH contributed equally to the response to the commentary. All authors contributed to the article and approved the submitted version.

## Conflict of Interest

The authors declare that the research was conducted in the absence of any commercial or financial relationships that could be construed as a potential conflict of interest.
